# Community ‘de-addiction’ camps: A follow-up study

**DOI:** 10.4103/0019-5545.46074

**Published:** 2005

**Authors:** Lok Raj, B.S. Chavan, Chandra Bala

**Affiliations:** * Senior Lecturer Department of Psychiatry, Government Medical College and Hospital, Chandigarh 160030; ** Professor and Head Department of Psychiatry, Government Medical College and Hospital, Chandigarh 160030; *** Medical Social Worker Department of Psychiatry, Government Medical College and Hospital, Chandigarh 160030

**Keywords:** Camp setting, detoxification, alcohol and drug dependence

## Abstract

**Aim:**

To evaluate the effectiveness of the camp approach for the treatment of alcohol and drug dependence.

**Methods:**

This was a prospective, longitudinal study with a 6-month follow-up. Patients were drawn from the villages covered under the community outreach programme of the Department of Psychiatry of the Government Medical College and Hospital, Chandigarh. Forty-six patients with a history of substance use (alcohol: 23, opioids: 20, cannabis: 2, sedative/hypnotic: 1) admitted to two different community camps for 10 days each were followed up in the community outreach clinics of the respective villages. Sociodemographic and clinical variables were recorded on a semi-structured proforma. Outcome variables, changes in dose, frequency, route of intake, status of complications, etc. were recorded on a specially designed semi-structured proforma at the end of 6 months of treatment. The ICD-10 criteria were used for diagnoses.

**Results:**

Thirty-six patients (78.3%) completed 6 months' follow-up, 61% recovered totally or their condition improved markedly. There were significant improvements in physical health and family life.

**Conclusion:**

The camp approach is a cheap and effective treatment alternative for patients with alcohol and drug dependence.

## INTRODUCTION

About 20 years ago, the famous Alma Ata conference of the World Health Organization emphasized the role of the community in the delivery of healthcare. The primary healthcare programme was the outcome of the Alma Ata Declaration. Although a large number of primary health centres (PHCs) and subsidiary health centres were opened in the rural sector, they failed to provide specialized care due to inadequate facilities. To boost healthcare in rural settings, a camp approach was started in which professionals reached the community at the doorstep. With the help of local resources, they started organizing camps for eye ailments, family welfare activities, school health, immunization, etc. These camps became successful and attractive. More and more medical problems are now being tackled through the camp approach. Drug and alcohol dependence is one area where patients do not prefer hospitals for various reasons.^1^ Also, community-based programmes for drug and alcohol dependence are almost non-existent. De-addiction camps have been organized sporadically in some parts of India, particularly in Jodhpur,^2^ around Chennai^3^ and Chandigarh.^1^

The camp approach has been advocated as an effective alternative to hospital-based treatment of drug and alcohol dependence, offering many advantages such as direct participation of the community in the treatment process, better acceptance by the patients, better compliance and cost-effectiveness.^1^ However, we still require more data and long-term follow-up for documenting the treatment outcome of the camp approach to support these claims. Ranganathan reported that of the 105 alcoholics treated in community de-addiction camps in Manjakkudi, a village in Tamil Nadu, between 1989 and 1992, 87 stayed sober after one year of follow-up.^3^ Successful treatment through the camp setting has also been reported from South-East Pacific Asian countries (Laos and Thailand).^4^ Reasonably good abstinence rates have been reported from Myanmar following treatment in camp settings.^5^

Compliance with treatment has been a problem with patients suffering from substance use disorders. In the USA, 30%–35% of patients do not complete treatment in hospital settings.^6^–^8^ Similarly, studies from India report a drop-out rate of 36%–69%.^9^,^10^ A high rate of treatment retention has been reported for the camp approach. Of the 20 patients treated by Chavan and Priti in a camp, no patient left treatment prematurely;^1^ whereas Purohit and Razdan reported low drop-out rates of 5%–10%.^2^

Longer follow-up has been reported in a few studies only. Ranganathan advocates 1-year follow-up as a part of the treatment.^4^ In her own study, she reports an abstinence rate of 85% after 1 year. Datta *et al*. report even higher rates of abstinence (93%).^11^ In both these studies only alcoholics were taken and in the latter, strict diagnostic criteria were used. The patients belonged to only one village in these studies. However, in terms of outcome, there are serious limitations in these studies. It is not mentioned what variables were considered to measure the outcome; the inferences seem to be largely impressionistic. The present study was carried out on patients treated in two different camps and the sample was heterogeneous. Apart from abstinence, changes in the dose, and frequency and status of complications were assessed to determine the outcome.

## METHODS

Preparations for each camp started 1 month in advance. During this period, the medical social worker met each patient identified with the help of local community leaders and local medical practitioners. Forty-six patients diagnosed according to the ICD-10 criteria were admitted in two different camps (23 in each camp) organized in the community for 10 days each. Patients with uncontrolled co-morbid psychiatric conditions were excluded. The first camp was organized in Palsaura village, situated at the outskirts of Chandigarh, during 11–20 November 1998 and the second camp was organized at Maloya village during 11–20 November 1999.

Patients with alcohol dependence were detoxified with benzodiazepines and vitamin supplements, and those with opioid dependence were detoxified with buprenorphine or dextropropoxyphene. Other medicines (non-steroidal anti-inflammatory drugs [NSAIDs], antidiarrhoeals, hypnotics, etc.) were used for symptomatic relief. After discharge, the patients were followed-up once a week in the community clinics of the respective villages by a team comprising a consultant psychiatrist, a medical social worker and a community nurse. The patients who dropped out were contacted through home visits by the social worker to ensure regular follow-up.

Thirty-six patients remained in the study at the end of 6 months of follow-up (78.3%). The sociodemographic variables of these 36 patients are given in Table 1. One patient died a few months after discharge from the camp because of respiratory infection and 1 patient was in police custody (details of both these patients are not available). Another patient was from a far-off village who had come regularly for the initial 3 months and was abstinent till then. Seven patients could not be contacted despite repeated attempts.

**Table 1:** Sociodemographic variables of the patients

	At baseline (*n*=46)	At 6 months (*n*=36)
		
Variables	*n*	*n*
Age		
Up to 20 years	2 (4.3)	0
21–40 years	29 (63.0)	23 (63.9)
41–60 years	14 (30.4)	12 (33.3)
61 years and above	1 (2.2)	1 (2.8)
Marital status		
Single	12 (26.1)	8 (22.2)
Married	31 (67.4)	27 (75.0)
Divorced	2 (4.3)	1 (2.8)
Widower	1 (2.2)	0
Occupation		
Skilled and semi-skilled labourer	32 (69.6)	25 (69.4)
Business/agriculture	8 (17.4)	6 (16.7)
Others	6 (13.0)	5 (13.9)
Education		
Up to matric (class X)	39 (84.8)	29 (80.6)
Above matric	7 (15.2)	7 (19.4)
Family type		
Nuclear	20 (43.5)	16 (44.4)
Joint	23 (50.0)	17 (47.2)
Others	3 (6.5)	3 (8.3)
Locality		
Urban	13 (28.3)	12 (33.3)
Rural	33 (71.7)	24 (66.7)

Values in parentheses are percentages.

A semi-structured proforma was prepared to record the outcome at the end of 6 months. The proforma recorded the change in drug use variables such as dose, frequency and route of administration, etc. and drug-related complications in physical, social, psychological, family, occupational and legal areas; apart from global subjective assessment by the reduction in the dose is also a sign of significant improvement, which was achieved in 50% of the patients (Table 2).

**Table 2:** Frequency of use and dose at six months (*n*=36)

Variables	*n*
Dose	
Total abstinence	8 (22.2)
Dose decreased significantly	18 (50.0)
No change	10 (27.8)
Frequency of use	
Total abstinence	8 (22.2)
Occasional use (once a month)	4 (11.1)
Once or twice a week	5 (13.9)
Daily but once only	9 (25.0)
Frequency same or increased	10 (27.8)

Values in parentheses are percentages.

## RESULTS AND DISCUSSION

The drop-out rate was 16.3%, which is far below the drop-out rate for hospital-based treatment (between 36% and 69%).^9^,^10^ If we take total abstinence as the main outcome criterion, the only 22.2% patients fulfill this. However, a 50%–75% reduction in the dose is also a sign of significant improvement which was achieved in 50% of the patients (Table 2). Thus patients who showed improvement and abstinence account for 72.7%, which is significant in view of the low cost of treatment. The rate of abstinence is less than the follow-up figures reported in earlier studies on the camp approach.^9^,^10^ The difference may be because of the design of the study. In both the above studies, no structured or semi-structured instrument was used to assess the outcome.

There was also a significant reduction in drug-related physical and familial complications (Table 3, p<0.01). Although an improvement was seen in social complications, the difference failed to reach a statistically significant level. However, in the majority of cases, there was no change in the legal and occupational status. This may be attributed to a longer period required for complete recovery, associated social stigma hampering job opportunities, lack of motivation and widespread unemployment.

**Table 3:** Clinical variables of the patients

	At baseline (*n*=46)	At 6 months (*n*=36)	
			
Variables	*n*	*n*	χ^2^/p value
Diagnosis			
Alcohol dependence	23 (50.0)	19 (52.8)	NS
Opioid dependence	20 (43.5)	15 (41.6)	NS
Cannabis dependence	2 (4.3)	1 (2.8)	NS
Sedative/hypnotic dependence	1 (2.2)	1 (2.8)	NS
Complications			
Physical	30 (65.2)	11 (30.6)	<0.01*
Familial	31 (67.4)	11 (30.6)	<0.01*
Social	22 (47.8)	13 (36.1)	NS
Occupational	27 (58.7)	22 (61.1)	NS
Legal	6 (13.0)	5 (13.9)	NS

* significant, NS: not significant

Values in parentheses are percentages.

About 61.1% of the patients perceived themselves as totally recovered or improved. The percentage agreement between the ratings by the patient himself and the family was high, i.e. up to 95% (Table 4). The outcome at 6 months was not influenced by sociodemographic variables (Table 5). Recovery from alcohol or drug dependence is a difficult and long process, and depends upon a number of factors such as medical, social and psychological management, social support, and rehabilitation facilities. Apart from professional inputs, it requires a change in the attitude of the family members in particular and the community at large. The camp approach is a cheap, feasible and effective alternative to hospital-based management of alcohol/drug dependence and paves the way for rehabilitation of these individuals because of the active involvement of the community in the treatment process.

**Table 4:** Global assessment at six months (*n*=36)

	Recovered and improved	No change	
			
Assessment by	*n*	*n*	Agreement
Patient	22 (61.1)	14 (38.9)	95%
Family	23 (63.9)	13 (36.9)	

Values in parentheses are percentages.

**Table 5:** Outcome relationship with sociodemographic variables (*n*=36)

Variable	Improved and recovered	No change	χ^2^
Age			
Up to 40 years	16	7	0.89
Above 40 years	7	6	NS
Marital status			
Married	17	10	0.17
Single (others)	6	3	NS
Occupation			
Labourers	17	10	0.17
Others	6	3	NS
Education			
Up to matric (class X)	20	9	1.14
Above matric	3	4	NS
Family type			
Nuclear	8	9	2.69
Non-nuclear	15	4	NS
Locality			
Urban	9	3	0.30
Rural	14	10	NS

NS: not significant

The results of this study are encouraging. A generalization may not be possible because of the small sample size. Further work can take direction from these results and more structured packages for detoxification and follow-up can be planned. A heterogeneous group (diagnostically) was another limitation, which caused problems in assessing the outcome by using standardized instruments. Separate diagnostic categories can be taken up for different camps to make the input more concentrated and goal-directed.
